# Hemi-Y incision for nipple-sparing mastectomy with immediate implant reconstruction: 10-year outcomes from a single-center series

**DOI:** 10.1016/j.jpra.2026.01.050

**Published:** 2026-02-13

**Authors:** Chloe Jordan, Krzysztof Sosnowski, Rushabh Shah, Sanjeev Hariparsad, William M. Nabulyato, Parto Forouhi, Charles M. Malata

**Affiliations:** a Department of Plastic and Reconstructive Surgery, Addenbrooke’s Hospital, Cambridge University Hospitals NHS Foundation Trust, Hills Rd, Cambridge, United Kingdom; b School of Clinical Medicine, University of Cambridge, Hills Rd, Cambridge, United Kingdom; c Cambridge Breast Unit, Addenbrooke’s Hospital, Cambridge University Hospitals NHS Foundation Trust, Hills Rd, Cambridge, United Kingdom; d Anglia Ruskin University School of Medicine, Anglia Ruskin University, East Rd, Cambridge, United Kingdom

**Keywords:** Hemi-Y incision, Nipple-sparing mastectomy, Implant-based breast reconstruction, Oncoplastic surgery, Long-term outcomes

## Abstract

**Introduction:**

Nipple-sparing mastectomy (NSM) is increasingly performed for both risk-reducing and therapeutic indications, with oncological outcomes comparable to conventional mastectomy and improved aesthetic outcomes. Several incision patterns have been described, with no single approach universally preferred. The hemi-Y incision combines a limited peri‑areolar component (25% of the circumference) with an inferior radial extension from 6 o’clock, providing adequate exposure for mastectomy and implant placement in appropriately selected cases, while partially concealing the scar along the nipple–areola complex (NAC).

**Materials and methods:**

We performed a retrospective review of all patients undergoing hemi-Y NSM with immediate implant-based reconstruction at a tertiary center between November 2009 and December 2019. Data collected included demographics, comorbidities, oncological treatments, operative details, peri‑operative complications, and long-term outcomes.

**Results:**

Eleven patients (20 breasts) underwent hemi-Y NSM with immediate implant reconstruction. The cohort comprised predominantly risk-reducing procedures, with one case of ductal carcinoma in situ and no invasive carcinomas. Complete 10-year follow-up was available for nine patients (18 breasts); two patients (4 breasts) were lost to follow-up. Early complications occurred in 3 breasts (15%): one superficial wound dehiscence, one seroma, and one infection with wound breakdown. There were no cases of nipple-areola complex (NAC) or mastectomy skin-flap necrosis, or peri‑operative implant loss. Over 10 years, three patients (15%) required implant revision or exchange, giving a 10-year implant survival rate of 89% (16/18 breasts). No local or NAC recurrences were observed. One patient developed brain metastases at 10 years, while all remaining patients were alive and disease-free.

**Conclusion:**

The hemi-Y incision provides adequate access for NSM and implant reconstruction with low complication rates and favorable scar concealment. Ten-year follow-up confirms durable reconstructive and oncological outcomes, supporting its role as a practical and reproducible option for patients undergoing NSM with implant reconstruction, predominantly in the risk-reducing and ductal carcinoma in situ (DCIS) setting. It should be considered in patients with small- to moderate-sized, non-ptotic breasts as a viable option for optimizing aesthetic outcomes following conservative mastectomy and implant-based reconstruction.

## Introduction

Breast cancer surgery has evolved from radical procedures to modern oncoplastic approaches that preserve oncological safety while improving cosmetic outcomes.^1^ Nipple-sparing mastectomy (NSM) is now widely used in risk-reducing surgery and selected cases of in-situ and invasive disease where the nipple–areola complex (NAC) can be preserved, with low recurrence rates.^2^

Several incision patterns have been described for NSM, aiming to balance oncological clearance, reconstructive access, and scar placement. Incisions are broadly categorized as extra-areolar or peri‑areolar (adjacent to the NAC),^3^ with neither approach proven superior. The hemi-Y incision, adopted by the senior authors (PF and CMM), combines a limited peri‑areolar incision (25% of the circumference) with a 6 o’clock radial extension. This provides adequate exposure for mastectomy and implant reconstruction, while partially concealing the scar along the NAC border. However, long-term outcome data remain limited.

## Methods

All patients undergoing hemi-Y nipple-sparing mastectomy (NSM) with immediate implant-based reconstruction at Addenbrooke’s University Hospital between November 2009 and December 2019 were retrospectively reviewed. Electronic records were used to collect demographic, operative, oncological and outcome data, with 10-year follow-up available for nine patients. Outcomes assessed included early complications, implant revisions, implant survival, and oncological status. All patients provided informed consent for surgery and inclusion of anonymized data.

## Surgical technique

**Figure 1:**
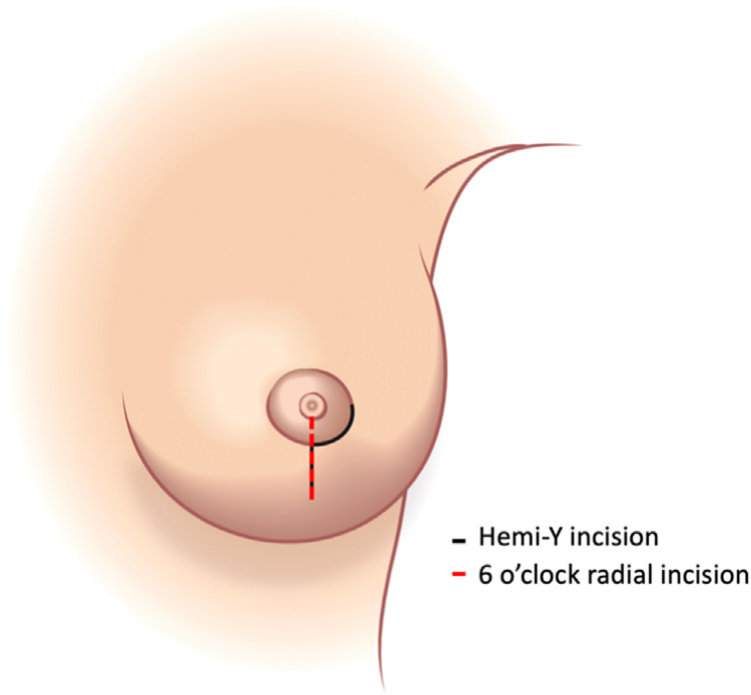
Schematic of the hemi-Y incision (black) compared with a standard 6 o’clock radial incision (red). The hemi-Y combines a limited peri‑areolar segment (approximately 25% of the areolar circumference) with an inferior radial extension, providing greater arc length than a radial incision alone (¼ circumference ≈ 1.57r vs radius = *r*). This design increases exposure for mastectomy and implant placement while preserving nipple–areola complex vascularity and concealing the scar within the areolar border.

With the patient upright, standard breast markings including the midline, meridian, inframammary fold are made. The hemi-Y incision consists of a peri‑areolar segment (approximately 25% circumference) with an inferior radial extension from 6 o’clock, which may be lengthened as required while preserving vascularity (Figure 1). Through this incision, a subpectoral pocket is fashioned and an implant or expander is placed.

## Results

Eleven patients (20 breasts) underwent hemi-Y NSM with immediate implant reconstruction (Table 1). Indications were predominantly risk-reducing; one patient had ductal carcinoma in situ (DCIS). No cases of invasive carcinoma were included. Exposure was adequate in all cases, with scars blending into a vertical mastopexy pattern.

**Table 1:** Demographics, operative details, and outcomes of patients undergoing hemi-Y nipple-sparing mastectomy with immediate implant reconstruction (*n* = 11 patients, 20 breasts). Data are presented as mean (range) or number (%). Early complications were limited to minor wound issues and seroma, with no cases of total nipple–areola complex (NAC) necrosis, flap loss, or peri‑operative implant loss. Long-term outcomes include implant survival, revisions, and oncological status at 10 years.

Variable	Result
Age, years	Mean 41 (range 25–54)
BMI, kg/m²	Mean 24.5 (range 21.3–35.3)
Indication	9 risk-reducing (82%), 1 DCIS (9%), 1 benign fibroadenomas (9%)
Laterality	8 bilateral (73%), 3 unilateral (27%)
Smoking status	3 smokers (27%), 8 non-smokers (73%)
Implant type	12 expandable (60%), 8 fixed-volume (40%)
ADM use	9/20 breasts (45%) (Surgimend or Strattice)
Early complications	3/20 (15%): wound dehiscence, seroma, infection
NAC or mastectomy flap necrosis	0
Peri-operative implant loss	0
Revisions/exchanges (10 yrs)	3 patients (15%)
10-year implant survival	89% (16/18 breasts with follow-up)
Local/NAC recurrence	0
Distant disease	1 patient (brain metastases at 10 yrs)

There were no cases of NAC or mastectomy skin-flap necrosis. Early complications occurred in 3 breasts (15%), including minor wound breakdown, infection, and seroma (Table 1). No peri‑operative implant losses were recorded. During 10-year follow-up, three patients (15%) required revision or implant exchange for capsular contracture, infection, or cosmetic reasons. All revisions were performed via the original hemi-Y scar.

Long-term oncological outcomes were favorable; no local or NAC recurrences were observed. One patient developed distant brain metastases at 10 years; the remainder were disease-free. Two patients were lost to late follow-up but included in peri‑operative analysis.

## Discussion

Nipple-sparing mastectomy (NSM) is increasingly adopted for prophylactic and therapeutic indications, offering oncological safety with improved aesthetic outcomes.^4^ The hemi-Y incision combines a short peri‑areolar limb with a 6 o’clock radial extension, designed to optimize access while preserving NAC vascularity and facilitating implant-based reconstruction.

The rationale for this design can be understood geometrically. One-quarter of the areolar circumference equals *1/4 × 2πr = 1.57r*, which exceeds the areolar radius (*r)*. Incorporating a short peri‑areolar component into a radial incision increases the available arc length for exposure without dividing the areola, allowing wider access for mastectomy and implant delivery while minimizing NAC devascularization. In contrast to intra-areolar scars, the periareolar component blends with the NAC border, while the inferior radial limb resembles a well-accepted vertical mastopexy scar.

Perfusion studies have demonstrated that inframammary fold incisions compromise NAC vascularity more than peri‑areolar approaches. By limiting the circumareolar component to approximately 25% of the NAC circumference, the hemi-Y incision aims to preserve perfusion while providing central access for reconstruction. Unlike longer circumareolar or lateral extensions, this configuration balances exposure, vascular preservation and scar concealment.^5^

While peri‑areolar incisions with extensions are well described, long-term outcome data for this limited-arc hemi-Y configuration are lacking. Most published series report short-term outcomes of 1–3 years. Our study provides the first 10-year follow-up of the hemi-Y incision, demonstrating low complication rates without NAC necrosis or peri‑operative implant loss. Over a decade, three patients required implant revision or exchange, yielding a 10-year implant survival of 89%. No local or NAC recurrences were observed, and one patient developed distant metastases at 10 years. The findings support the long-term reconstructive durability and oncological safety of the hemi-Y incision.

Importantly, the hemi-Y incision should not be presented as a universal oncologic approach. In our series, the majority of procedures were risk-reducing, with one case of ductal carcinoma in situ and no invasive carcinomas. Based on these data, the hemi-Y is best suited to small-to-moderate, non-ptotic breasts undergoing prophylactic NSM or selected DCIS, where central exposure and implant delivery are priorities and scar concealment is desired.

This study has limitations, including its small cohort size and retrospective design. The inferior radial extension is not primarily designed for superior pole tumors or axillary access; in such cases, alternative incisions or a separate axillary approach may be preferable. The limited requirement for axillary surgery in this series reflects the predominance of risk-reducing procedures. Within these constraints, the hemi-Y incision represents a safe, reproducible option within a defined NSM reconstructive niche.

## Conclusion

The hemi-Y incision provides reliable access for nipple-sparing mastectomy and implant placement while concealing the scar along the NAC border. The inferior radial component follows a familiar vertical mammoplasty scar pattern. Our 10-year follow-up confirms durable reconstructive outcomes, low complication rates, and no local or NAC recurrences. The hemi-Y incision represents a practical and reproducible option for appropriately selected patients, particularly those undergoing risk-reducing procedures or selected cases of DCIS.

## Funding

None.

## Declaration of competing interest

None declared.
